# The Discoloration effect of White Mineral Trioxide Aggregate (WMTA), Calcium Enriched Mixture (CEM), and Portland Cement (PC) on Human Teeth

**DOI:** 10.4317/jced.54075

**Published:** 2017-12-01

**Authors:** Amin Salem-Milani, Saeede Ghasemi, Saeed Rahimi, Amir Ardalan-Abdollahi, Mohammad Asghari-Jafarabadi

**Affiliations:** 1 Assistant Professor , Dental and Periodontal Research Center, Tabriz University of Medical Sciences, Tabriz, Iran; 2 Dentist, Private practice; 3 Professor of Endodontics, Dental and periodontal research center, Tabriz University of Medical Sciences, Tabriz, Iran; 4 Assistant Professor, Department of Endodontics, Dental School, West Azarbaijan, Urmia University of Medical Sciences, Urmia, Iran; 5 Associate Professor of Biostatistics, Department of Statistics and Epidemiology, Faculty of Health, Tabriz University of Medical Sciences, Tabriz, Iran

## Abstract

**Background:**

The aim of this study was to evaluate the discoloration induced by CEM cement, Portland cement (PC) and MTA mixed with propylene glycol (MTA-PG) in comparison to White MTA.

**Material and Methods:**

Ninety extracted premolar and canine teeth were resected 2 mm below the CEJ. The coronal part of crown was prepared with peeso reamer and Gates-Glidden drills, and the specimens were randomly divided into 4 experimental (n=20) and one control (n=10) groups. The tooth crowns in experimental groups 1 to 4 were filled with White MTA, PC, CEM cement and MTA-PG, respectively; and in group 5, the teeth were kept empty. After incubation, digital photographs of teeth were acquired at 4 time points (before, immediately after placing the materials, 3 and 6 months afterwards). Images were transferred to Adobe Photoshop CS4 and CIE L*a*b color space was used for tooth shade assessment. One-Way ANOVA and One-Sample t-test were used to compare discoloration of teeth between groups.

**Results:**

Significant statistical discoloration was only observed in the cervical one third of all groups at each time points (except between 3 and 6 months). Tooth discoloration was greatest in PC and lower in MTA and MTA-PG at the end of 6 months. The tooth discoloration between immediately and 3 months after placing the materials had significant difference only between MTA and PC; and also the tooth discoloration between immediately and 6 months after placing the materials was observed only between PC and MTA, and PC and MTA-PG.

**Conclusions:**

All of the experimental biomaterials caused tooth discoloration after 6 months, of those, PC had the most and MTA and MTA-PG had the least discoloration effect.

** Key words:**Mineral trioxide aggregate (MTA). Calcium enriched mixture (CEM). Propylene glycol. Portland cement.

## Introduction

Since the introduction of Mineral trioxide aggregate (MTA) as a retro-filling material by Torabinejad (1) in 1995, MTA has been widely used in different clinical situations due to numerous favourable properties such as high biocompatibility and low cytotoxicity (2), sealing ability (3), release of calcium hydroxide (Ca(OH)2)(4), antibacterial effect (5), reasonable compressive strength, and acceptable hardness (5,6). Despite these advantages, MTA has shown some drawbacks such as long setting time, difficult handling, and tooth color change (6-8). 

Tooth and gingival discoloration due to clinical applications such as direct pulp capping, full or partial pulpotomy (9), sealing of coronal perforations (10) , root fractures and root resorptions treatments (11) and revascularizations of immature teeth (12) is a major MTA disadvantage (6). Although white MTA (WMTA) has been developed to overcome the tooth discoloration caused by the application of grey MTA (13), several *in vivo* and *in vitro* studies have also reported tooth discoloration after using WMTA (14). Poor handling is another drawback of MTA (15).

To improve the characteristics of MTA, changes have been made in its powder or liquid (16). One of the materials mixed with MTA in order to enhance its handling is propylene glycol (PG) (15,16). PG is an alcoholic, colorless and odorless compound without toxicity, carcinogenicity or genotoxicity. Also, it has hygroscopic characteristics (17,18). Studies have shown that adding PG to MTA improves its flow and push-out bond strength without any adverse effect on the biocompatibility of MTA (15,16). Furthermore, mixing MTA with PG, increases the release of Ca(OH)2 and consequently its pH (17). However its effect on discoloration of MTA is not clear.

Recently, a new biomaterial called calcium enriched mixture (CEM) has been developed. Desirable physical and biological characteristics and high clinical success of CEM cement has been demonstrated (19). In addition to advantages of MTA, CEM has shown shorter setting time and better handling (3,19). The chemical composition of MTA and CEM cement is different (19) and no studies have been published about the effect of CEM on tooth color. 

Portland cement (PC) is the main composition of MTA (20). Various studies have demonstrated the similar physical and biological characteristics of MTA and PC (21,22). The only difference between MTA and PC is the existence of bismuth oxide in the mixture of MTA (20), which has been recently introduced as the main reason of tooth discoloration of MTA (23). Therefore, discoloration of teeth due to application of PC may be theoretically lower than MTA (14).

Since no studies have compared the tooth color change due to application of CEM, PC and MTA-PG, the aim of this study was to evaluate the discoloration induced by these biomaterials in comparison to White MTA.

## Material and Methods

This study was approved by the Research and Ethics Committee of Tabriz University of Medical Sciences. Ninety extracted human canine and premolar teeth extracted for periodontal or orthodontic reasons were selected for this study. All the teeth had mature single straight roots, with no caries or restorations, no hypoplasia or severe attrition or tooth color change. In addition, the teeth with cracks or fractures and pulp chamber or canal calcifications were excluded. Following extraction, the external root surface was cleaned with periodontal curette following polishing with pumice powder and water, and the teeth were stored in 3% chloramine-T solution at 4°C. The roots were resected 2 mm apical to the cement enamel junction with a diamond disk (D&Z, Darmstadt, Germany) and water spray. For preparation of pulp chamber Hedstrom files (Mani, Japan) and #2-5 Gates-Glidden drills (Dentsply, Maillefer, Ballaigues, Switzerland) and finally #6 peeso reamer (Mani, Japan) were used. 5 ml of 2.5% NaOCl (Taj Corp, Tehran, IRI) was used for irrigation. The smear layer was removed using 2 mL of 17% ethylenediaminetetraacetic acid (EDTA) (Pulpdent Corp., Watertown, MA, USA) for 2 min, followed by a rinse with five mL of 5.25% NaOCl for 2 min. Finally, 10 mL of normal saline was used as final irrigation and the paper cones were used for drying canals. The 90 samples were randomly divided into four experimental groups (n=20) and one control group (n=10) so that the number of each tooth type (canine and premolar) was similar in groups. In group 1, White MTA powder (Angelus,Londrina,Brazil) was mixed with distilled water by the ratio of 1 g powder to 0.33 mL liquid. In group 2 and 3, CEM Cement (BioniqueDent, Tehran, Iran) and white PC (Tehran Cement Co., Tehran, Iran) powders were mixed with distilled water by the ratio of 1 g powder to 0.33 mL liquid. In group 4, 1g powder of White MTA (Angelus, Londrina, Brazil) was mixed with 0.33 mL PG (Merck, Germany) and distilled water (20% PG and 80% distilled water by volume). These materials were prepared according to the manufacturer’s directions and placed into the teeth crowns up to the CEJ using MTA carrier and packed with an endodontic plugger (size 9/11; Hu-Friedy, Chicago, IL). Then cotton pellets moistened with distilled water were placed over the materials and the apical ends were sealed with sticky wax (Dentsply Tulsa Dental, Johnson City, TN). The samples were incubated at 100% humidity in an incubator at 37°C for 24 hours. After that, the wet cotton pellets were removed from the canals and the roots were sealed with sticky wax again and incubated at the same conditions for 6 months. The blindness of the color change evaluator was provided by allocating a code to each tooth.

-Tooth Shade Assessment

For tooth color measurement, a digital photography was taken from buccal aspects of teeth in four time points: T1: before material placement, T2: immediately after material placement, T3: 3 months after material placement and T4: 6 months after material placement. The images were taken using a high resolution digital camera (Canon EOS 600D/ 18 MP Canon Inc., Taiwan) under standardized lightening conditions. The images were transferred to Adobe Photoshop CS4 (Adobe, San Jose, CA) software, and CIE l*a*b* system was used for color assessment in incisal, middle and cervical one thirds of tooth crowns. For this purpose, an area of 51×51 pixels was determined in the middle of each one third areas and the rate of color change was recorded. Casmatch (Bear Medic Corp, Tokyo, Japan) shade reference cards were included in each image to provide a reference for shade control during analysis(7). In CIE l*a*b* system the amount of “L” ranges from 0 (black) to 100 (white) and indicates the rate of lightness. “a” and “b” represent the color change in green-red and blue-yellow axes, respectively. The discoloration was calculated using following formulation (24): ∆E=√(“(∆l)²+(∆a)²+(∆b)²” ).

-Statistical Analysis

Statistical analysis was performed using SPSS software (SPSS version 16.0, SPSS, Chicago, IL, USA). One-way ANOVA and Tukey Post-hoc tests were used to compare the rates of ∆E among the groups (in different locations). One-Sample t-test was used to evaluate the color change of groups at different time points in comparison with the standard amount detectable by the human eye (3.7) (25). The level of significance was set at 0.05.

## Results

**Figure 1:**
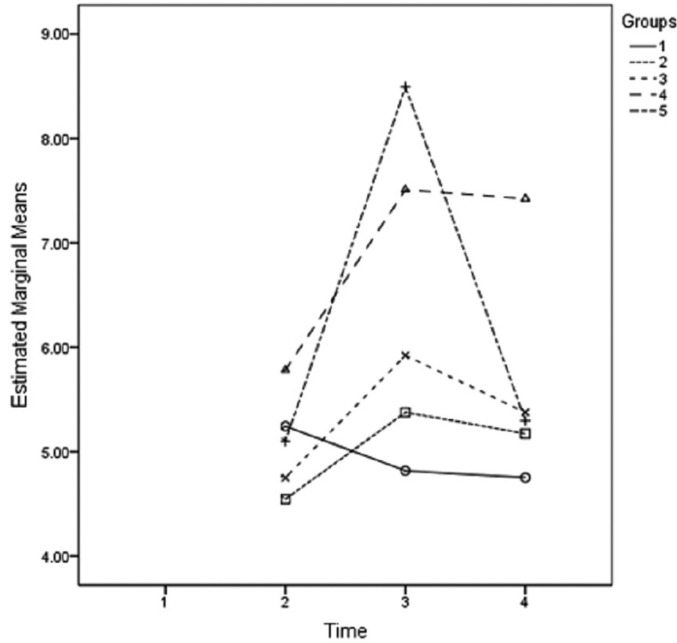
Mean color changes (ΔΕ) at different groups and different times.

There was no significant difference among the groups regarding the rate of ∆E in incisal and middle one thirds of teeth (*p*<0/05). In cervical one thirds of all groups, the color differences between before and after material placement (*P*=0.007), immediately after and 3 months after material placement (*P*=0.013), immediately after and 6 months after material placement (*P*=0.001) were significant. However, the color difference between 3 months and 6 months after material placement was not significant. According to the results of post-hoc Tukey test, the color change between before and after material placement was only significant between MTA-PG and PC groups (*P*=0.002). Also the color change between T2 and T3 was only significant between MTA and PC groups (*P*=0.017). Furthermore, the color change of T2 and T4 was significant between PC and MTA groups (*P*=0.002) and PC and MTA-PG (*P*=0.001) (Fig. 1).

## Discussion

The results of the present study showed that the significant color change, which was distinguishable by the human eyes, could be only observed in cervical one thirds of teeth. This finding is in accordance with the study of Partovi *et al.* (26). The reason of different color change of cervical and incisal one thirds may be the different thickness of dentin in those parts. Also, the thinner thickness and colorless structure of enamel in the cervical region causes the color change more obvious (26). Furthermore, this study showed that 3 months after material placement, the greatest color change was in PC, and MTA showed the least discoloration, which was significantly lower than PC group. This minimal color change was hardly differentiated by the human eyes. This finding collaborates with the study of Ioannidis *et al.* (8) Also, Ioannidis *et al.* (27) in another study concluded that application of MTA Fillapex did not cause significant color change. Furthermore, Felman and Parashos (7) demonstrated that WMTA induced the color change of tooth crown and the effect was compounded in the presence of blood. Moreover, the comparison of crown tooth color, 6 months after material placement, revealed that PC had the most color change, whereas MTA and MTA-PG showed the least color change which was significantly lower than PC and not significantly different from control group. This finding is in contrast with the results of Lenherr *et al.* (14) which showed that the lowest color change related to PC after 12 months. The reason of this contrast is not clear and more studies are necessary. The other finding of this study was the slight color change observed in the control group in all of the time periods. Since it was tried to make all of the temperature, humidity and imaging conditions the same in this study, the reason of the color change was not clear and needs further investigations. As stated previously, mixing PG with MTA improves its handling properties and increases the pH, Ca(OH)2 release, setting time, flow and push-out bond strength over time. In the present study, the mixture of 80% distilled water and 20% PG with MTA was selected, since it was the best recommended proportion (17,18). According to the results of this study, although the color change of MTA and MTA-PG were significant after 6 months, their difference was not significant, and they showed the least rate of discoloration among the study groups. Previous studies have attributed the discoloration effect of MTA to the presence of bismuth in its mixture (23,28). Since the amount of bismuth in the PC is much less than MTA (29), previous researches concluded that the rate of color change due to use of PC should be lower than MTA (14,30). However, in this study, the rate of color change due to the application of PC in all of the time periods was higher than other materials. According to the simulation of all of the experimental conditions of previous studies, the reason of this color change is not clear. The results of the present study demonstrated that use of CEM cement resulted in tooth discoloration visible by human eyes which increased over time. Compounds including FeO, MgO and Al2O3 were lower in CEM in comparison to MTA. Also, there was not any bismuth oxide in the mixture of CEM. If the color change of MTA is attributed to these mixtures especially bismuth oxide, there will be no known reason for the increased rate of color change of CEM compared with MTA. The present study showed the least discoloration effect of the MTA and MTA-PG in comparison to other materials. Further studies are recommended with the use of spectrophotometry, large sample sizes and long-term follow ups in order to overcome the controversies.

## Conclusions

According to the results of this study, all of the experimental biomaterials caused tooth discoloration after 6 months. Of these, PC had the most, and MTA and MTA-PG had the least discoloration effect.
